# Construction and validity of the Interprofessional Communication in Health Scale

**DOI:** 10.1590/0034-7167-2022-0483

**Published:** 2023-08-07

**Authors:** Cláudia Patrícia da Costa Brás, Manuela Maria Conceição Ferreira, Maria do Céu Aguiar Barbieri de Figueiredo

**Affiliations:** I Instituto de Ciências Biomédicas Abel Salazar. Porto, Portugal; II Escola Superior de Enfermagem de Coimbra. Coimbra, Portugal; III Escola Superior de Saúde de Viseu, Instituto Politécnico de Viseu. Viseu, Portugal; IV Universidade de Huelva. Huelva, Espanha; V Centro de Investigações em Tecnologias e Serviços de Saúde & Rede de Investigação em Saúde. Porto, Portugal

**Keywords:** Communication, Patient Care Team, Nursing, Validation Study, Psychometrics., Comunicación, Grupo de Atención al Paciente, Enfermería, Estudio de Validación, Psicometría., Comunicação, Equipe de Assistência ao Paciente, Enfermagem, Estudo de Validação, Psicometria.

## Abstract

**Objectives:**

to construct and validate the Interprofessional Communication Scale in Health.

**Methods:**

a psychometric study was carried out on a sample of 360 nurses from a hospital and university center in central Portugal. Reliability was assessed through internal consistency and construct validity through exploratory and confirmatory factor analysis.

**Results:**

the Interprofessional Communication in Health Scale, consisting of 27 items, is organized into 3 factors: “Teamwork”, “Conflict management” and “Leadership”, with a total variance of 51.1%. Good internal consistency was obtained, with a Cronbach’s alpha of 0.842, and adequate Goodness of Fit Index model.

**Conclusions:**

the Interprofessional Communication in Health Scale presents a factorial structure with adequate validity and reliability results, and may constitute a useful self-report instrument in assessing interprofessional communication in health.

## RESUMO

**Objetivos:**

construir e validar a Escala de Comunicação Interprofissional em Saúde.

**Métodos:**

o estudo psicométrico foi realizado em uma amostra com 360 enfermeiros de um centro hospitalar e universitário da região centro de Portugal. A confiabilidade foi avaliada através da consistência interna e a validade do construto pela análise fatorial exploratória e confirmatória.

**Resultados:**

a Escala de Comunicação Interprofissional em Saúde, constituída por 27 itens, está organizada em 3 fatores: “Trabalho em equipe”, “Gestão de conflitos” e “Liderança”, com uma variância total de 51,1%. Obtiveram-se boa consistência interna, com alfa de Cronbach de 0,842, e índices adequados de qualidade de ajustamento do modelo.

**Conclusões:**

a Escala de Comunicação Interprofissional em Saúde apresenta uma estrutura fatorial com resultados adequados de validade e de confiabilidade, podendo constituir-se um instrumento de autorresposta útil na avaliação da comunicação interprofissional em saúde.

## RESUMEN

**Objetivos:**

construir y validar la Escala de Comunicación Interprofesional en Salud.

**Métodos:**

el estudio psicométrico se realizó sobre una muestra de 360 enfermeras de un centro hospitalario y universitario del centro de Portugal. La confiabilidad se evaluó a través de la consistencia interna y la validez de constructo mediante análisis factorial exploratorio y confirmatorio.

**Resultados:**

la Escala de Comunicación Interprofesional en Salud, compuesta por 27 ítems, se organiza en 3 factores: “Trabajo en equipo”, “Gestión de conflictos” y “Liderazgo”, con una varianza total del 51,1%. Se obtuvo una buena consistencia interna, con un alfa de Cronbach de 0,842, y adecuados índices de bondad de ajuste del modelo.

**Conclusiones:**

la Escala de Comunicación Interprofesional en Salud presenta una estructura factorial con resultados adecuados de validez y confiabilidad, pudiendo constituir un instrumento de autoinforme útil en la evaluación de la comunicación interprofesional en salud.

## INTRODUCTION

Communication processes in the hospital environment are complex and dynamic, due to the enormous flow of information, the different teams of professionals and numerous highly complex interventions^(1)^. We continue to witness work overload due to lack of human resources, lack of standardization of conduct, inexperience of professionals and lack of leadership, factors that can lead to failures in communication and jeopardize the care provided to patient^(2-4)^. The persistence of hierarchical practices among different professionals, conflicts at work, difficulties in understanding the other’s role, do not favor teamwork and influence the way interprofessional communication is established^(1,4-6)^.

Health professionals inevitably work together, but are trained to work separately, which constitutes a barrier to the development of collaborative practices and effective teamwork^(5)^. It is imperative to break with the uniprofessional culture, with a stereotyped view of what a professional team is, which does not automatically translate into collaboration between professionals^(7)^.

Effective interprofessional communication favors continuous care, minimizing the occurrence of errors and contributing to patient safety^(8)^. However, it is essential that professionals, regardless of the category to which they belong, make shared decisions, build knowledge in a dialogical way, taking into account the vision and mission of hospital organization^(2,6,9)^. Dialogue should be understood as a tool to consolidate interprofessional communication, which is recognized as the capacity for effective communication between people, especially from different professions, and considered essential to achieve collaborative interprofessional practice in health^(2,10)^.

Teamwork, interprofessional conflict resolution and collaborative leadership are essential domains for promoting effective interprofessional collaboration^(10)^. In this perspective, it becomes relevant to invest in training models in health proposed by the World Health Organization, pointing to integrated and interactive learning, with structured information systems and processes, effective communication strategies, policies for conflict resolution and frequent dialogues between the teams^(5,11-12)^, where differences of opinion should be assumed as constructive interactions^(9)^.

The role of leading nurses is essential in the development of teamwork, capable of influencing collective work and multidisciplinary interaction for better health outcomes^(13)^. This leader’s commitment presupposes an authentic leadership process, implying self-knowledge about their strengths, limitations, coherence in its actions, transparency in the sharing of information and a role of influencing others through its proactive, ethical and responsible behavior in order to build an environment of trust and integrity in health services^(14-15)^.

Effective communication with assertive behavior when sharing information, an interpersonal relationship anchored in collaboration, respect and mutual help, has an impact not only on patient safety, but also on professionals’ professional and personal scope^(3,9)^, being considered one of the international goals of patient safety, established by the Joint Commission International in partnership with the World Health Organization.

Communication skills cannot be improved only with clinical experience; they must be trained, improved, validated, to promote changes in health professionals’ attitude and behavior, with a beneficial effect on their self-efficacy and with the aim of improving health service quality^(16)^.

In the literature review, no instruments were found that would allow an assessment of interprofessional communication in health, in some of the competence domains referenced by the Canadian Interprofessional Health Collaborative. Thus, it is understood the importance of developing a self-completion instrument that will allow data on interprofessional communication skills to be obtained. Thus, the study aimed to build and validate the Interprofessional Communication in Health Scale (ECIpS - *Escala de Comunicação Interprofissional em Saúde*).

## OBJECTIVES

To construct and validate the Interprofessional Communication in Health Scale.

## METHODS

### Ethical aspects

The study was conducted in accordance with international ethics guidelines and approved by the ethics committee integrated in the innovation and development unit -clinical trials center and by the board of directors of the hospital and university center in the central region of Portugal, obtaining formal authorization for study continuation. Carrying out the study safeguarded, at different times of the process, the ethical and deontological principles enshrined in the Declaration of Helsinki and in the Portuguese legislation in force that governs research with human beings (Decree Law 80/2018). Free and informed consent was obtained from all participants involved in the study, expressed in writing.

### Study design, period, and place

This is a psychometric study with nurses from a hospital and university center in central Portugal. The research took place from September 2018 to May 2019. The COSMIN (consensus-based standards for the selection of health measurement instruments) protocol was used for the research design, in accordance with the EQUATOR network^(17)^.

### Population or sample; inclusion and exclusion criteria

The sample of this study is non-probabilistic for convenience, consisting of 360 nurses. The sample calculation was performed taking into account the criteria for carrying out the factor analysis, respecting the ratio of 10:1 (number of respondents for each item of the questionnaire), to obtain stable factor solutions^(18)^. As inclusion criteria, all nurses who provided direct care to users at the hospital and university center in the central region of Portugal were considered. The exclusion criteria established were performing functions as a nurse manager and being temporarily absent from the service during the data collection period, due to a medical certificate, vacation leave or other leave.

### Study protocol

The construction of this scale was supported by an exhaustive literature review, the competence domains highlighted by the National Interprofessional Competency Framework^(10)^ and the teaching-learning objectives identified in the Health Professions Core Communication Curriculum (HPCCC) of the European Association for Communication in Health Care (EACH)^(19)^, which can allow assessing clinical communication according to the institutional needs and specificities and/or professional groups.

In an initial phase, the items that allowed measuring the construct under study were defined, obtaining a provisional version of an instrument consisting of 40 items. Next, we proceeded to content validity, using two procedures: judges’ analysis and semantic analysis. The panel of judges consisted of a psychometrist, two professors and a clinical practice nurse, experts in the area of communication, who assessed language clarity, item practical and theoretical pertinence^(20)^, having defined as a criterion to incorporate the items with agreement between judges above 75%^(21)^. Regarding the Content Validity Coefficient (CVC) per judge, this was between 0.777 and 0.883. The final calculation of CVC with polarization factor was 0.830. The validity ratio coefficient was determined using the Lawshe model adjusted by Tristan^(22)^, for content validity quantitative verification, given that the number of panelists is less than 5. The minimum acceptable for the adjusted validity ratio is 0.582. In this study, six of the items were eliminated, as they presented values below the acceptable value for the validity ratio in all measured criteria, there was no inter-judge agreement. We also found that three items on the scale had values of 0.5 in the theoretical relevance criterion. However, the value close to the minimum acceptable for theoretical relevance, adequate values in the other two criteria and the intention to subsequently carry out an exploratory and confirmatory analysis, allowed us to assume these three items for the final scale.

The consensus version consisted of 34 items, being submitted to a pre-test with 17 nurses from clinical practice, where the study would take place, in order to analyze item understanding, clarity and applicability. All modifications and suggestions presented were incorporated into the final version of the scale. Participants were asked to respond to each item on a 5-point scale, between “never” (1) and “always” (5), intending to assess the theoretical dimensions of communication in health teams, with higher values revealing better interprofessional communication skills in health. The final instrument, called the Interprofessional Communication in Health Scale (ECIpS - *Escala de Comunicação Interprofissional em Saúde*), also included a questionnaire that allowed the sociodemographic characterization.

### Analysis of results, and statistics

Data analysis was performed using the software IBM^®^ SPSS^®^ Statistics for Windows, version 27.0 (IBM Corp., Armonk, N.Y., USA), and the AMOS^®^ 27 software (Analysis of Moment Structures)^(23)^.

Reliability studies configured the determination of item internal consistency or homogeneity through Pearson’s correlation coefficient (correlations greater than 0.20), determination of Cronbach’s alpha coefficient, considering good internal consistency values greater than 0.80^(24)^ and determination of McDonald’s omega coefficient, which considers acceptable values of 0.70 and 0.90 and good internal consistency values greater than 0.90^(25)^.

To extract common factors from item interpretation, an exploratory factor analysis (EFA) was applied. EFA applicability was verified through: (i) the Kaiser-Mayer-Olkin coefficient (KMO> 0.5); (ii) Bartlett’s sphericity test (p< 0.05); and (iii) the main diagonal values of the anti-image matrix of correlations with a measure of sample adequacy greater than 0.5. The principal components (PC) method was used to reduce the original items to a lower number of common factors, based on three aspects: Kaiser criterion (eigenvalues greater than 1); scree plot criterion; and total extracted variance (50% is the minimum acceptable value). The process of interpreting the extracted factors was optimized using orthogonal rotation (Varimax) of the axes.

In the confirmatory factor analysis (CFA), the model was estimated using the maximum likelihood method, previously assessing the assumptions of normality through the coefficients of asymmetry (Sk<=3), kurtosis (Ku<= 7) and Mardia’s multivariate coefficient (< 5)^(26)^. Overall Goodness of Fit Indexes were taken into account, such as the ratio between chi-square and degrees of freedom (x^2^/df), considering a good fit values lower than 2-3, acceptable, if lower than 5, and unacceptable, if higher than 5^(27)^. Goodness of Fit Index (GFI) and Comparative Fit Index (CFI) values equal to 1 are indicators of a model with perfect fit to the data; values greater than 0.95 indicate an optimal fit; values between 0.90 and 0.95 point to a good fit; and values below 0.9 indicate a poor fit^(28)^. However, we can consider values between 0.8 and 0.9 indicating a model with a poor fit to the data^(29)^. Regarding the Root Mean Square Error of Approximation (RMSEA), optimal adjustments are considered when values are considered lower than 0.05, good, for values between 0.05 and 0.08, and unacceptable, for values greater than 0, 08^(30)^. A 90% confidence interval (CI) is calculated for the RMSEA, considering that an upper limit of 90% CI of less than 0.1 is indicative of a good fit^(29)^. Standardized Root Mean Square Residual (SRMR) values are considered good when less than 0.08, considering a perfect fit when root mean (RMR)=0^(31)^. In the model adjustment, modification indices greater than 11, proposed by AMOS, were considered^(23)^.

The local adjustment quality was carried out through factorial weights (λ) greater than 0.50, which may be relaxed to 0.40 in exploratory psychometric studies, due to individual item reliability (r2) with coefficients equal to or greater than 0.25 and the composite reliability (CR), which considers good internal consistency factors with values above 0.70^(29,32)^. One of the conditions for considering the existence of a higher-order hierarchical factor is the existence of strong correlations (correlation values close to 1) and statistically significant between first-order factors, the existence of at least three first-order factors and a conceptual support that support the existence of the factors^(33)^.

Convergent validity was assessed by average variance extracted (AVE), discriminant validity by comparing AVE with Pearson’s correlation square, and construct reliability assessed by CR. Reference values were considered to be AVE>0.5, CR≥0.7 and existence of discriminant validity when the squared correlation between the factors is lower than the AVE for each factor^(26,29)^.

## RESULTS

### Sample characteristics

The sample under study consists mostly of female nurses (82.8%), with an average age of 42 years. Most nurses are married or living in a stable relationship (63.6%). Predominantly, 78.6% of nurses hold a degree in nursing as academic qualifications, and 38.3%, as specialist nurse. On average, nurses have 19 years of professional experience. Of the participants, 5.3% participated in training courses on communication techniques and communication in health teams.

### Interprofessional Communication in Health Scale psychometric property analysis

To validate the ECIpS psychometric quality, reliability and validity studies were carried out. In an initial phase, the reliability value was calculated for the total of the 34 items that make up the scale. With regard to item-total correlation coefficients, most items showed good correlation rates, with the exception of items 7.30 and 31, which are below the critical value of 0.20 defined as the minimum limit. Cronbach’s alpha coefficients per item were analyzed again, and were found to be above 0.8, with an overall Cronbach’s alpha of 0.876 after eliminating the three items^(34)^ and a McDonald’s omega value of 0.928^(25)^. Thus, the EFA proceeded based on this structure of items that meet the initial criteria to remain in the statistical procedure. The average values and respective standard deviations of the different items, as a whole, are well centered, being above the expected average index according to Table 1.

**Table 1:** Internal consistency of the Interprofessional Communication in Health Scale items (N=360), Coimbra, Beira Litoral, Portugal, 2018-2019

Items		M^*^(±SD^**^)	Total r/item	α^***^ without item 1^st^ assessment	α ^***^ without item 2^nd^ assessment	Ω ^* ***^ MacDonald
1	I have an attitude of affection and solicitude towards team members	4.07 (±0.61)	0.512	0.837	0.871	0.929
2	I demonstrate a positive attitude to motivate others	4.04 (±0.61)	0.597	0.835	0.869	0.929
3	I relate to team members in a delicate and caring way	4.14 (±0.60)	0.582	0.835	0.870	0.929
4	I show empathy and concern in the relationship	4.14 (±0.60)	0.642	0.835	0.869	0.928
5	I establish cooperative relationships. assertiveness and trust with team members	4.15 (±0.54)	0.630	0.835	0.870	0.928
6	I develop and maintain relationships based on truth	4.48 (±0.58)	0.479	0.837	0.871	0.930
7	I develop my thinking about other team members	4.18 (±2.22)	0.137	0.856	----	----
8	I understand and respect the individuality and roles of each team member	4.29 (±0.62)	0.485	0.837	0.872	0.930
9	I consider the needs and interests of team members	4.06 (±0.61)	0.459	0.837	0.870	0.930
10	I understand the diversity of teams, skills and professional knowledge, and I take advantage of this for the benefit of all	4.00 (±0.62)	0.546	0.836	0.876	0.929
11	I provide opportunities for interaction and opinion formation within the group	3.83 (±1.24)	0.296	0.840	0.871	0.932
12	I accept criticism and listen to divergent ideas/perspectives as stimulators of creativity and innovation	4.03 (±0.60)	0.522	0.836	0.869	0.929
13	I create conditions for the divergence of perspectives to be channeled towards improving the quality of care	3.79 (±0.68)	0.611	0.834	0.882	0.929
14	I allow team members opportunities to mobilize their skills in the development of care	3.94 (±1.66)	0.226	0.845	0.873	0.933
15	I express my own interests in a simple and direct way, distinguishing them from the team goals	3.38 (±0.92)	0.345	0.838	0.869	0.932
16	I have the ability to negotiate within the healthcare team based on compromise (mutual interests)	3.58 (±0.78)	0.563	0.834	0.870	0.929
17	I can effectively manage conflict by channeling it towards improving the quality of care	3.66 (±0.69)	0.567	0.835	0.893	0.929
18	I value my skills in problem-solving	3.92 (±2.27)	0.214	0.852	0.869	0.933
19	I reveal effective participation in the decision, planning and coordination of care	3.83 (±0.67)	0.605	0.834	0.870	0.929
20	I share knowledge and skills	4.07 (±0.56)	0.606	0.835	0.892	0.929
21	I help foster team spirit to achieve better results	4.17 (±2.24)	0.216	0.852	0.869	0.933
22	I have the ability to integrate team members	4.08 (±0.64)	0.580	0.835	0.869	0.929
23	I facilitate consensus building	3.87 (±0.65)	0.602	0.835	0.870	0.929
24	I recognize the commitment and continued commitment of the team members	3.92 (±0.61)	0.571	0.836	0.870	0.929
25	I obtain elements that allow me to understand the impact of my actions and the way in which they are being received and interpreted by others	3.64 (±0.65)	0.539	0.836	0.869	0.930
26	I review carefully and in detail all the information I convey to team members	3.72 (±0.72)	0.598	0.834	0.869	0.929
27	I provide team members with indications of how they are or are not succeeding in delivering care	3.46 (±0.81)	0.565	0.834	0.870	0.929
28	I inform the team about my perceptions, thoughts and needs	3.59 (±0.77)	0.528	0.835	0.870	0.930
29	I am clear and concise in sharing messages with the healthcare team	3.92 (±0.63)	0.570	0.835	0.871	0.929
30	I provide relevant information to guide team members	3.97 (±1.72)	0.209	0.846	---	---
31	I transmit to the other team members the relevant information that users transmit to me	4.34 (±2.17)	0.157	0.854	----	----
32	I have scientific knowledge to present data about patients and their clinical details to team members	4.00 (±0.61)	0.498	0.837	0.871	0.930
33	I keep clear and adequate records (written or electronic) of meetings	3.99 (±0.73)	0.401	0.838	0.869	0.931
34	I refer people/institutions to help solve problems	3.65 (±0.80)	0.401	0.837	0.870	0.931

* M - mean;

** SD - standard deviation;

*** α - Cronbach’s alpha; Ω^****^ - McDonald’s omega.

The factor analysis applicability was verified using the Kaiser-Mayer-Olkin coefficients (KMO = 0.930), which allows classifying it as excellent by Bartlett’s sphericity test (p < 0.000) and by the values of the main diagonal of the anti-image matrix of correlations with measures of sampling adequacy greater than 0.5, which allows proceeding with the factor analysis^(24)^.

The initial factorial solution presented a structure with 7 factors that together explained 59.67% of the total variance. The proportion of the variance of each variable explained by the factors (commonality) is within the reference values (0.40) when they oscillate between 0.43 and 0.76. However, the scree plots graph configured the existence of three factors, so a new factorial analysis was carried out, forcing three factors. All factors have factor loadings in items greater than 0.40, except items 11, 14, 18 and 21, so these items were excluded and a new extraction was performed. The final factorial solution explains 51.1% of the total variance after rotation. Factor 1 was called “Teamwork” and explains 37.9% of the total variance, consisting of items 1, 2, 3, 4, 5, 6, 8, 9, 10 and 12. Factor 2, “Conflict management”, comprises items 13, 15, 16, 17, 23, 24, 25, 27 and 28 and explains 8.4% of the total variance. Factor 3 was called “Leadership” and consists of items 19, 20, 22, 26, 29, 32, 33 and 34 and explains 4.8% of the total variance, as shown in Table 2.

**Table 2:** Matrix of principal components after Varimax rotation of the 27 items (N=360), Coimbra, Beira Litoral, Portugal, 2018-2019

Items		Factors		
	1	2	3	Initial ^*^h^2^
5	0.81			0.73
3	0.79			0.69
4	0.78			0.72
1	0.71			0.60
2	0.69			0.66
8	0.67			0.66
6	0.60			0.56
9	0.59			0.57
10	0.51			0.52
12	0.46			0.43
16		0.72		0.62
15		0.71		0.56
28		0.66		0.66
27		0.66		0.60
25		0.63		0.64
17		0.55		0.52
13		0.52		0.53
24		0.46		0.59
23		0.46		0.55
33			0.70	0.60
32			0.66	0.51
34			0.53	0.61
22			0.52	0.64
20			0.50	0.60
19			0.49	0.58
26			0.45	0.55
29			0.42	0.47

* *h2 - commonalities.*

**Figure 1:**
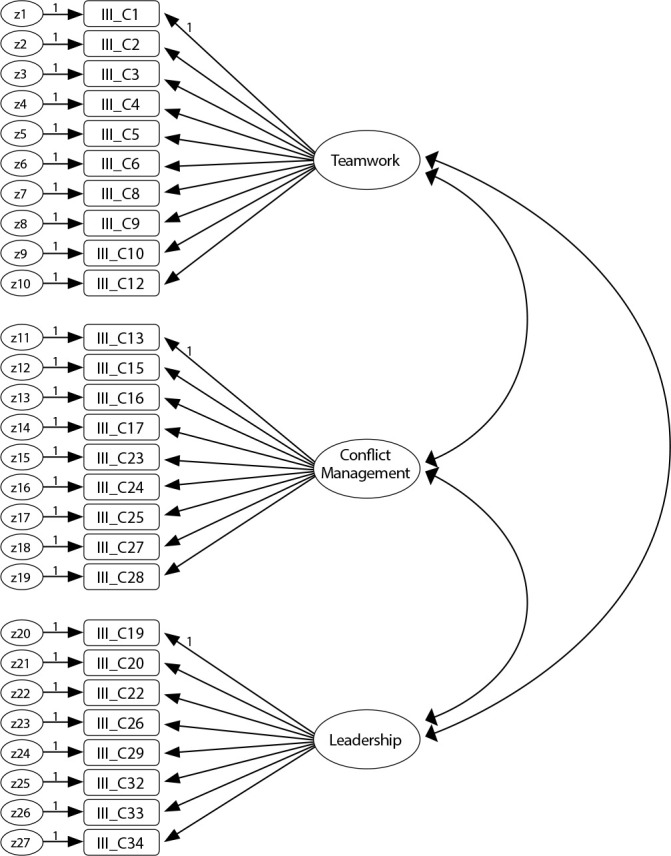
Initial model with 27 items, Coimbra, Beira Litoral, Portugal, 2018-2019

After analyzing item sensitivity, it was observed that, in general, the values of asymmetry and kurtosis do not compromise the CFA’s performance^(26)^, since in absolute values they oscillate between 0.04 and 0.94 for asymmetry and between 0.03 and 2.46 for kurtosis. Mardia’s multivariate coefficient is 4.17. These values fall short of the reference values. The critical ratios of the paths between latent and manifest variables are statistically significant, leading to item maintenance. Figure 1 presents the four-factor model, where the 27 items distributed by the corresponding factors are observed as well as the respective factor weights and their individual reliability. It is visible that item IIIC15 of factor 2 and items IIIC33 and IIIC34 of factor 3 have saturations and individual reliability lower than recommended, which is why they will be eliminated. In the initial model (Figure 1), most of GFI were adequate, x2/df=2.85, for RMR=0.02, SRMR= 0.06 and RMSEA=0.07 (with upper limit of 90%CI of 0.077), with the exception of GFI=0.83, CFI=0.87, which revealed a poor fit.

**Figure 2:**
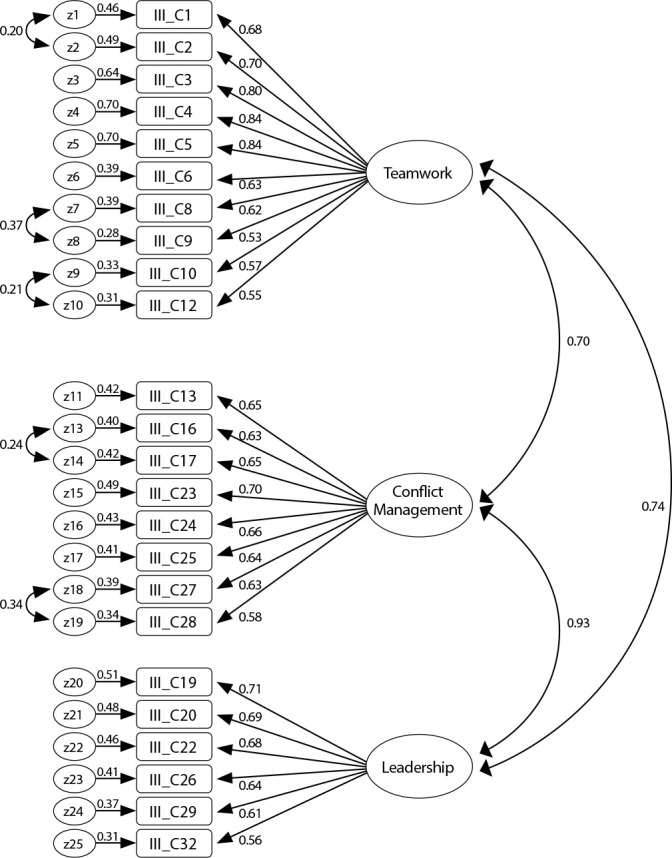
Model with deleted items and modification indexes, Coimbra, Beira Litoral, 2018-2019

The model was refined using the modification indices (MI), as shown in Figure 2. After assessing the theoretical plausibility of modifications, the measurement errors that led to the improvement of the measurement adjustment were correlated, with the exception of the GFI index =0.87, which remains in a poor fit.

**Figure 3:**
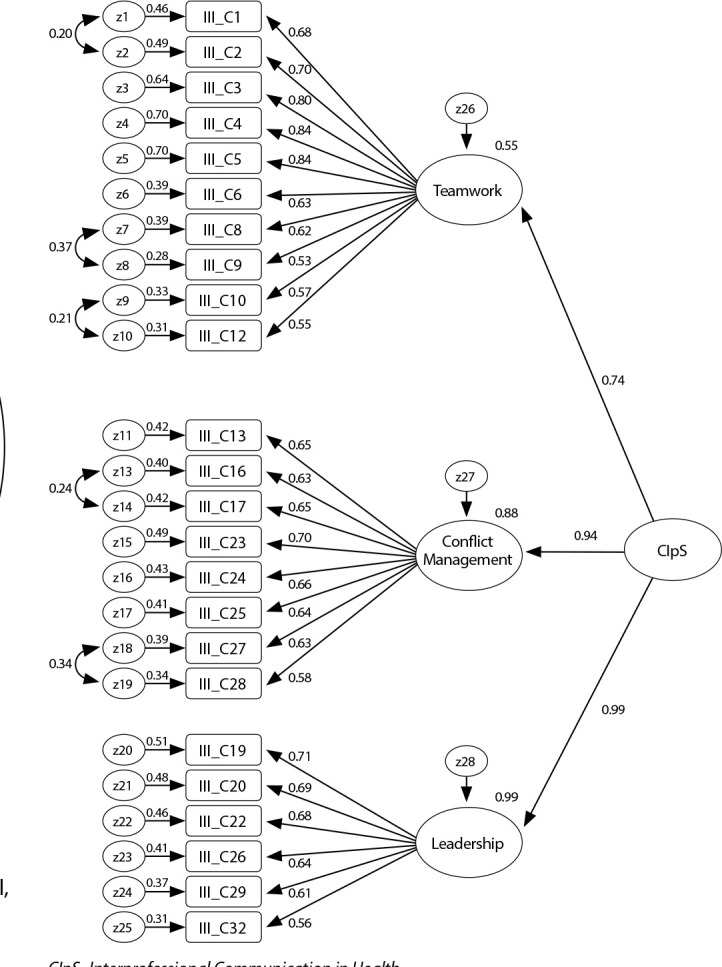
2^nd^ order model, Coimbra, Beira Litoral, Portugal, 2018-2019

All factors considered to be 1^st^ order are positively and significantly correlated (“Teamwork” and “Conflict management” (r=0.70; p<0.01), “Conflict management” and “Leadership” (r= 0.93; p<0.01) and “Teamwork” and “Leadership” (r=0.70; p<0.01)). These correlations suggest the existence of a hierarchical structure with a second-order factor called Interprofessional Communication in Health (CIpS). Figure 3 illustrates the obtained model, noting that the highest correlational intensity of the CIpS construct is verified with the “Leadership” dimension (r=0.99; p<0.01) followed by the “Conflict management” dimension (r=0.99; p<0.01). =0.94; p<0.01) and “Teamwork” (r=0.74; p<0.01).

*CIpS- Interprofessional Communication in Health*

The following overall GFI were obtained for the second-order model: x2/df=2.48; RMR=0.02; SRMR=0.05; RMSEA=0.06 (90%CI upper limit of 0.071); GFI=0.87; and CFI=0.91. The CR of the factors proved to be adequate, being 0.9 for the factor “Teamwork” and “Conflict management” and 0.8 for the factor “Leadership”^(32)^. Convergent validity, obtained through AVE, was not confirmed, since all factors had indexes below 0.50. The discriminant validity is only evident between the factors F1_Teamwork_ vs F3_Leadership_=0.55^(29)^. The study of this scale is concluded, verifying that the correlations are positive and statistically significant (p<0.01), with all factors being strongly associated with its overall dimension (2^nd^ order factor). On the other hand, it appears that the factors are significantly associated with each other in a reasonable to moderate way, with values ranging between r=0.61 and r=0.77. The relationship between F2_Conflict Management_ and F3_Leadership_ with r(358)=0.77, p<0.01, and F1_Teamwork_ with F3_Leadership_ r(358)=0.67, p<0.01 stands out.

## DISCUSSION

The use of self-completion instruments allows the development of understanding, reflective, critical and creative thinking^(35)^. The construction and validity of instruments that measure communication skills in health teams are of great interest, as they are fundamental tools for health professionals who strive for excellent care.

The ECIpS generally presented good indicators of reliability and validity, as verified by the results obtained. The sample with 360 participants has a ratio of 10.58 respondents per item, giving robustness to the results obtained^(18)^. Regarding the scale reliability, the results show good item internal consistency and homogeneity. An overall Cronbach’s alpha coefficient of 0.876 was found, which is classified as adequate^(26)^ and a McDonald’s omega of 0.928^(25)^. The KMO of 0.930 and Bartlett’s sphericity test with a statistically significant probability (p=0.000) were indicative of continuing with the exploratory factor analysis^(36)^. Carrying out the factorial analysis, using the PC method, with Varimax-type orthogonal rotation and eigenvalues greater than 1, allowed explaining 51.1% of total variance, involving a theoretically acceptable number of factors and constituting the best solution in relation to the interpretation and meaning of dimensions^(37)^.

Submitting this four-factor structure to CFA, using the maximum likelihood method, led to the elimination of some items, after the respecification of the model, because they present multicollinearity problems and saturations below 0.40, considered the minimum coefficient, since this is a preliminary study of the scale^(29)^.

The final second-order model revealed adequate composite reliability indices and overall GFI, with the exception of GFI index=0.87, which remains in a poor fit^(29)^. In this regard, it is recommended in the future to replicate the psychometric study in larger samples to obtain greater sensitivity and also to carry out parallel analyzes to confirm this factorial structure in a more in-depth way using other types of software such as FACTOR^®^ or statistical program R^®^.

Overall, the scale consisting of 24 items organized into three factors, “Teamwork”, “Conflict management”, and “Leadership”, demonstrates to be able to assess interprofessional communication in health.

### Study limitations

The study limitations are related to the fact that the sampling technique is non-probabilistic for convenience, limiting the generalization of results. An assessment was not carried out through direct observation, which would avoid the influence of the critical and reflective capacity of those who assess themselves.

### Contributions to nursing and health

This multidimensional assessment instrument will allow obtaining data on interprofessional communication skills in health, which support training interventions, in order to encourage collaborative interprofessional practice. This instrument, which assesses key dimensions for communication in health teams, will contribute to improving health team practices, anchored in effective, cohesive and consolidated communication.

## CONCLUSIONS

Health organizations are spaces of strong interaction between different professionals. In this regard, interprofessional communication in a clinical context is fundamental, considering the need for safe and effective responses in health care provision. It was in this sense that the ECIpS was created and the present psychometric study is justified. This scale consists of 24 items organized into three dimensions: Teamwork; Conflict management; and Leadership.

The study of ECIpS psychometric characteristics makes it possible to state that it is a robust instrument and certifies its quality and the theoretical relevance of each item included in the three dimensions that constitute it. ECIpS constitutes a valuable resource for assessing interprofessional communication skills in health, sensitizing nurses and other health professionals to this topic, in order to build the necessary collaborative interfaces to better converge in response to health challenges.

We suggested that more studies be carried out at a national level with the scale replication, obtaining increasingly better model adjustment indices.

## Data Availability

https://doi.org/10.48331/scielodata.JAZSA3
